# O.4.2-3 Physical activity participation among disabled people: secondary analysis of the 2018/19 Active Lives survey

**DOI:** 10.1093/eurpub/ckad133.178

**Published:** 2023-09-11

**Authors:** Shelby Carr, Andrew Atkin, Karen Milton

**Affiliations:** University of East Anglia, UK; s.carr@uea.ac.uk; University of East Anglia, UK; s.carr@uea.ac.uk; University of East Anglia, UK; s.carr@uea.ac.uk

## Abstract

**Purpose:**

Disabled people face significant barriers to being physically active and typically have low levels of activity. To enhance both access to and engagement in physical activity, it is valuable to understand the activity choices of disabled people. This study describes the types and duration of physical activity participation among disabled people and compares this to participation among non-disabled people.

**Methods:**

This study used data from the 2018/19 sweep of Sport England’s Active Lives survey, a nationally representative assessment of activity levels in adults living in England. From a prespecified list of 212 activities, participants reported their participation in the previous 12 months (yes/no) and duration in the previous four weeks (min/week). For this study, activities were subsequently collapsed into 17 mutually exclusive groups, which included ‘leisure activities’ (for example, walking, and gardening) and ‘invasion games’ (for example, football and rugby). Participants also reported whether they had a long term (>12 months) health condition and whether this affected their normal daily activities; this informed the classification of participants into three groups: limiting disability, non-limiting disability, and no disability.

**Results:**

Data were available for 171,121 participants, of whom 31,666 (18.5%) had a limiting disability, 34,955 (20.4%) a non-limiting disability, and 104,500 (61.1%) no disability. Leisure activity was the most common activity type and had the highest median mins/week of activity (median, 315 [IQR = 120-705] limiting disability; median, 390 [IQR = 180-750], non-limiting disability; median, 379 [IQR = 165-750] no disability). Across the three participant groups, walking, swimming, and gym-based activity were among the most common activities undertaken. Compared to participants with no disability, participants with a limiting disability reported lower engagement and durations for all activity types, except motorsports.

**Conclusions:**

The types of activities undertaken by disabled people were largely similar to that of non-disabled people, but engagement and duration was typically lower. The necessary next step is understanding how to improve adoption of and adherence to different activity types among disabled people.

**Support/Funding Source:**

This research was funded by a University of East Anglia Faculty of Medicine and Health Studentship.

